# The attenuated end of the phenotypic spectrum in MPS III: from late-onset stable cognitive impairment to a non-neuronopathic phenotype

**DOI:** 10.1186/s13023-019-1232-0

**Published:** 2019-11-12

**Authors:** Stephanie C. M. Nijmeijer, L. Ingeborg van den Born, Anneke J. A. Kievit, Karolina M. Stepien, Janneke Langendonk, Jan Pieter Marchal, Susanne Roosing, Frits A. Wijburg, Margreet A. E. M. Wagenmakers

**Affiliations:** 1 Amsterdam UMC, Pediatric Metabolic Diseases, Amsterdam Lysosome Center “Sphinx”, University of Amsterdam, H8–264, Meibergdreef 9, Amsterdam, The Netherlands; 2 The Rotterdam Eye Hospital, Rotterdam, The Netherlands; 3 Erasmus MC, Department of Clinical Genetics, University Medical Center Rotterdam, Rotterdam, The Netherlands; 4 Salford Royal NHS Foundation Trust, Adult Inherited Metabolic Disorders, Mark Holland Metabolic Unit, Salford, UK; 5 Erasmus MC, Center for Lysosomal and Metabolic disease, Department of Internal Medicine, University Medical Center Rotterdam, Rotterdam, The Netherlands; 6 Amsterdam UMC, Psychosocial Department, Amsterdam Public Health Research Institute, University of Amsterdam, Amsterdam, The Netherlands; 7 Radboud University Medical Center, Donders Institute for Brain, Cognition and Behaviour, Department of Human Genetics, Nijmegen, The Netherlands

**Keywords:** Mucopolysaccharidosis type III, Sanfilippo syndrome, Phenotypic spectrum, Neuropsychology assessment, Learning difficulties

## Abstract

**Background:**

The phenotypic spectrum of many rare disorders is much wider than previously considered. Mucopolysaccharidosis type III (Sanfilippo syndrome, MPS III), is a lysosomal storage disorder traditionally considered to be characterized by childhood onset, progressive neurocognitive deterioration with a rapidly or slowly progressing phenotype. The presented MPS III case series demonstrates adult onset phenotypes with mild cognitive impairment or non-neuronopathic phenotypes.

**Methods:**

In this case series all adult MPS III patients with a mild- or non-neuronopathic phenotype, who attend the outpatient clinic of 3 expert centers for lysosomal storage disorders were included. A mild- or non-neuronopathic phenotype was defined as having completed regular secondary education and attaining a level of independency during adulthood, involving either independent living or a paid job.

**Results:**

Twelve patients from six families, with a median age at diagnosis of 43 years (range 3–68) were included (11 MPS IIIA, 1 MPS IIIB). In the four index patients symptoms which led to diagnostic studies (whole exome sequencing and metabolomics) resulting in the diagnosis of MPS III; two patients presented with retinal dystrophy, one with hypertrophic cardiomyopathy and one with neurocognitive decline. The other eight patients were diagnosed by family screening. At a median age of 47 years (range 19–74) 9 out of the 12 patients had normal cognitive functions. Nine patients had retinal dystrophy and 8 patients hypertrophic cardiomyopathy.

**Conclusion:**

We show the very mild end of the phenotypic spectrum of MPS III, ranging from late-onset stable neurocognitive impairment to a fully non-neuronopathic phenotype. Awareness of this phenotype could lead to timely diagnosis and genetic counseling.

## Introduction

In recent years, clinical and diagnostic studies have demonstrated that the phenotypic spectrum of many rare lysosomal storage disorders is much wider than previously thought. Mucopolysaccharidosis type III (MPS III or Sanfilippo syndrome), an autosomal recessive lysosomal storage disorder which is primarily characterized by progressive neurocognitive deterioration, is nowadays divided in a rapidly progressing and slowly progressing phenotype [1]. MPS III is caused by a deficiency of one of four enzymes involved in the stepwise degradation of the glycosaminoglycan (GAG) heparan sulfate (HS) [2]. Four different subtypes of MPS III are recognized (MPS IIIA-D), all resulting in HS accumulation in the central nervous system (CNS) triggering a secondary pathophysiological cascade with neuronal inflammation, apoptosis, astrocytosis, microgliosis and synaptic disorganization [3, 4]. Classical MPS III is clinically divided in three disease phases [1]. After an initial symptom-free phase, a developmental delay is generally noted at the age of 2–6 years. During the second phase, progressive loss of cognition, behavioral and sleeping problems manifest. During the third phase, generally starting in their teens, progressive motor deterioration results in complete dependency and loss of ambulation [5]. Most patients demise in their second or third decade of life [6]. Somatic disease is typically limited but may involve recurrent ear-, nose- and throat- (ENT) disorders, femoral head necrosis, hepatomegaly and, recently reported, subclinical cardiac abnormalities [5, 7, 8]. A characteristic sign of MPS III are the dysmorphic features including progressive facial coarsening with prominent eyebrows and hair, a protruding philtrum, and in some synophrys and hypertrichosis.Although all patients generally follow the same disease course, patients with a more attenuated, slowly progressing, phenotype have been described over the last decades [9–11]. In MPS IIIA (OMIM #252900) homozygosity for the missense mutations c.897C > T, p.(Ser298Pro) and c.617G > C, p.(Arg206Pro) in the sulfamidase (*SGSH*) gene resulted in an attenuated phenotype with a later onset of regression, a slower progression of neurocognitive decline, and a longer survival [12–14]. An attenuated phenotype has also been reported in patients with MPS IIIB (OMIM #252920) due to the missense changes p.(Arg643Cys), p.(Ser612Gly), p.(Glu634Lys), p.(Leu497Val) with stable intellectual disability for many years [10, 15]. Furthermore, two case reports previously reported three patients with an even more attenuated phenotype, presenting with cardiomyopathy, retinitis pigmentosa and adult-onset dementia [16, 17].

In this manuscript we report a multi-center case series of MPS III patients with a mild- or non-neuropathic phenotype defined as having completed regular secondary education and maintaining a level of independency during adulthood, further delineating the very mild end of the phenotypic spectrum, beyond the slowly progressing phenotype.

## Methods

All adult MPS III patients attending the outpatient clinic of one of three expert centers for lysosomal storage disorders, with a mild- or non-neuropathic phenotype were included in this case series. This mild- or non-neuropathic phenotype was defined as: 1) a completed regular secondary education and 2) independency during adulthood, involving either independent living or a paid job.

Diagnosis of MPS III was confirmed by investigation of urinary GAG levels (total or HS), enzymatic activity in leucocytes and/or fibroblasts and mutation analysis. Patient data were obtained from the following centers: Amsterdam University Medical Centers (Amsterdam UMC), The Rotterdam Eye Hospital, Erasmus Medical Center (all in the Netherlands), and Salford Royal NHS Foundation Trust (United Kingdom). Educational level was divided into three categories: low (primary, lower vocational, lower and middle general secondary education), intermediate (middle vocational, higher general, pre-university education) and high (higher vocational education and university) [18].This study was presented to the medical ethical committee of the Amsterdam UMC who declared that this study needed no ethical approval since this study entails a retrospective and anonymized chart review. Written informed consent for publication of the case histories and the photographs was obtained from all patients and, if legally obliged, from their parents and/or legal representatives.

In addition, the literature was reviewed for patients with a mild neuronopathic phenotype fulfilling the above criteria, in order to present a complete overview.

## Results

### Patient characteristics

Twelve patients from six families were included in this case series (Table 1). The median age at inclusion was 47 years (range 19–74). Median age at diagnosis was 43 years (range 3–68). Four patients were males (33%). Eleven patients were diagnosed with MPS IIIA and one with MPS IIIB.

**Table 1:** MPS III patients with a mild- or non-neuropathic phenotype

ID	Age at Diagnosis (years)	Current Age (years)	Symptom leading to diagnostic studies	MPS III Subtype ^a^	Mutation 1	Protein 1	Mutation 2	Protein 2	Enzyme Activity	Reference Range (nmol/mg.17 h)	Material	Urinary GAGs	Reference Range (mg/mmol creatinine)	Age at Decline (years)
1.1	64	65	RD	A	c.734G > A	p.(Arg245His)	c.545G > A	p.(Arg182His)	0.1	3–12	Leucocytes	HS: 110.5	0–7.6	
2.1	56	56	Family screening	A	c.734G > A	p.(Arg245His)	c.545G > A	p.(Arg182His)	0.1	3–12	Leucocytes	HS: 100	0–7.6	
3.1	62	62	Family screening	A	c.734G > A	p.(Arg245His)	c.545G > A	p.(Arg182His)	0	3–12	Leucocytes	HS: 10.1	0–7.6	
4.1	51	52	Family screening	A	c.734G > A	p.(Arg245His)	c.545G > A	p.(Arg182His)	0	3–12	Leucocytes	HS: 52.9	0–7.6	
5.1	53	54	Family screening	A	c.734G > A	p.(Arg245His)	c.545G > A	p.(Arg182His)	0.1	3–12	Leucocytes	HS: 70.9	0–7.6	
6.2	5	21	Family screening	A	c.892 T > C	p.(Ser298Pro)	c.1262C > G	p.(Thr421Arg)	0.1	4.1–10.7	Leucocytes	Total: 28	1–8	
7.2	3	19	Family screening	A	c.892 T > C	p.(Ser298Pro)	c.1262C > G	p.(Thr421Arg)	0.1	4.1–10.7	Leucocytes	Total: 23	5–15	
8.3	49	50	RD	A	c.1130G > A	p.(Arg377His)	c.545G > A	p.(Arg182His)	0.2	3–12	Leucocytes	HS: 134.6	0–7.6	
9.3	41	41	Family screening	A	c.1130G > A	p.(Arg377His)	c.545G > A	p.(Arg182His)	0	3–12	Leucocytes	HS: 25.1	0–7.6	
10.4	27	32	Family screening	B	c.1927C > T	p.(Arg643Cys)	c.1834A > G	p.(Ser612Gly)	0.0	0.70–2.60^b^	Leucocytes	HS: 6125	0–343	32
11.5	41	42	Sudden decline in neurocognitive functioning	A	c.220C > T	p.(Arg74Cys)	c.1063G > A	p.(Glu355Lys)	0.2	3.2–20	Leucocytes	Total: 13.6	0–8	41
12.6	68	74	HCM	A	c.734G > A	p.(Arg245His)	c.545G > A	p.(Arg182His)	0.2	20–90	Fibroblasts	Total: 19.7	0–5.2	
Cases from literature														
1 [16]	53	NK	Cardiomyopathy	A	NK	NK	NK	NK	0.6	1.1 – 12^c^	Leucocytes	Total: 5.3	2.4–4.8	
2 [17]	42	NK	Retinitis pigmentosa + dementia	C	NK	NK	NK	NK	4.1	13–46	Leucocytes	↑	NK	NK
3 [17]	46	NK	Retinitis pigmentosa + dementia	C	NK	NK	NK	NK	1.4	13–46	Leucocytes	↑	NK	NK

Abbreviations: *GAGs* glycosaminoglycans; *HCM* hypertrophic cardiomyopathy; *HS* heparan sulfate; *NK* not known; *RD* retinital dystrophy; *↑* increased heparan sulfate.

ID = This column depicts twelve patients (numbers 1–12 before the punctuation) from six different families (numbers 1–6 after the punctuation).

^a^ Gene per subtype = Type A: *SGSH*; type B: *NAGLU*; type C: *HGSNAT*.

^b^ Enzyme activity reference range is in nmol/mg.hr.

^c^ Enzyme activity reference range is in pmol/min/mg

### Symptom leading to diagnostic investigations

In the six families, the index patients initiating the diagnostic tests were retinal dystrophy in two index patients, hypertrophic cardiomyopathy (HCM) in one and a decline in neurocognitive functioning in three, of which two had a classical progressive phenotype and are therefore not included in this cohort. The diagnostic investigation leading to diagnosis was whole exome sequencing (WES) in 4 families and metabolic studies in the other 2 families.

### Metabolic studies

Urinary GAG levels were increased in all patients, and enzymatic activity in leucocytes or fibroblasts was markedly decreased and clearly within the patients range in all patients, confirming the diagnosis of MPS III in all twelve patients.

### Missense variants

In total, 7 different missense changes in the *SGSH* gene were reported of which 5 have previously been reported as pathogenic (p.(Ser298Pro), p.(Arg74Cys), p.(Glu355Lys), p.(Arg245His), p.(Arg377His)) [13, 19–21]. The other two variances of unknown significance are likely pathogenic based on in-silico analysis (p.(Arg182His): PhyloP 5.13 (conserved), CADD 22.9 (damaging, cut-off> 20) and p.(Thr421Arg): PolyPhen-2 0.98 (probably damaging, cut-off > 0.8), M-CAP 0.225 (damaging, cut-off >.025), CADD 20.7 (damaging, cut-off > 20), Provean −3.39 (damaging, cut-off −2.5), LRT (deleterious), MutationTaster (damaging)) [22]. Two missense changes in the *NAGLU* gene were found, both previously reported as pathogenic (p.(Arg643Cys) and p.(Ser612Gly)) [23, 24].

### Cases from the literature

Three cases with a comparably mild phenotype were previously reported in the literature, presented in Table 1. Mutation analysis was not mentioned for these patients.

### Neurocognitive testing

Neurocognitive testing had been recently performed in 8 of the 12 patients reported here, and were reported in 2 of the 3 patients reported in the literature (Table 2).

**Table 2:** Neurocognitive test results in MPS III patients with a mild- or non-neuropathic phenotype

Patient	Age at Testing (years)	Test	Domain	Index scores	95% CI
1.1 ^a^	64	WAIS-IV short form	VCI	111	105–116
			PRI	85	75–94
2.1 ^a^	55	WAIS-IV short form	VCI	93	88–99
			PRI	125	116–131
5.1 ^a^	54	WAIS-IV short form	VCI	100	94–106
			PRI	116	107–122
6.2 ^c^	20	WAIS-IV	FSIQ ^b^	79	75–85
			VCI	109	103–114
			WMI	80	74–89
			PRI	72	66–81
			PSI	59	54–73
7.2	18	WAIS-IV	FSIQ ^b^	96	91–101
			VCI	113	107–118
			WMI	92	85–100
			PRI	100	92–108
			PSI	76	69–88
8.3	50	WAIS-IV short form	VCI	93	88–99
10.4 ^c^	26	WAIS-III	FSIQ ^b^	68	65–73
			VCI	79	74–85
			WMI	68	63–77
			PRI	61	56–72
			PSI	55	51–69
11.5 ^c^	42	WAIS-IV	FSIQ	50	47–55
			VCI	66	62–73
			WMI	55	51–64
			PRI	52	48–61
			PSI	50	47–63
Cases from literature					
1 [17]	31	WAIS	FSIQ	88	NK
2 [17]	36	WAIS	FSIQ	75	NK

Neurocognitive test scores are based on a mean of 100 with a SD of 15.

Abbreviations: *FSIQ* full scale IQ; *NK* not known; *PRI* perceptual reasoning index; *PSI* processing speed index; *VCI* verbal comprehension index; *WAIS* Wechsler Adult Intelligence Scale; *WMI* working memory index.

^a^ VCI and PRI estimated using the proration method [25], extrapolating the scores on 2 subtest within each index.

^b^ FSIQ should be interpreted with caution given significant discrepancies between the index scores for the different components.

^c^ Functions with a mild neurocognitive impairment.

### Brief clinical history and highest attained educational level and achievements of patient cohort

#### Family 1

In this family 5 siblings are affected. Patient 1 is the index patient. Patients 2–5 were diagnosed by family screening.

##### Patient 1

**Fig. 1:**
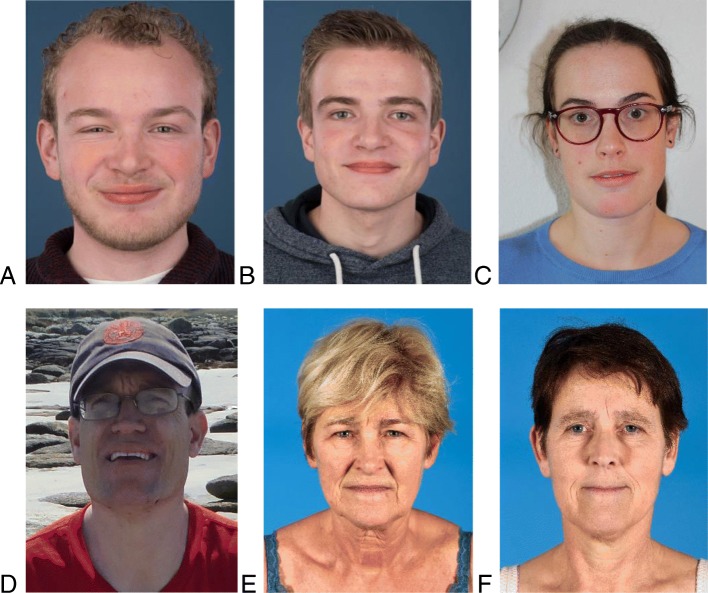
Mucopolysaccharidosis type III patients without typical or very mild dysmorphic features. **a**. Type IIIA; 21 years. **b**. Type IIIA, 19 years. **c**. Type IIIB; 32 years. **d**. Type IIIA; 42 years. **e**. Type IIIA; 65 years. **f**. Type IIIA; 56 years

This female patient was diagnosed with MPS IIIA by WES, at the age of 64 after evaluation for retinal dystrophy. Retinal dystrophy could not otherwise be explained. She has a completely normal cognitive functioning (Table 2). After the diagnosis, additional follow-up revealed an asymptomatic severe left ventricle hypertrophy (LVH) with a good cardiac function. Her highest completed educational level is middle vocational education (intermediate education level). She is currently 65 years old and performed the financial administration of her husband’s company until her recent retirement. She is the mother of three healthy children. She has her driver’s license, but can no longer drive due to loss of vision. She has no dysmorphic features (Fig. 1e).

##### Patient 2

Patient 2 was diagnosed at the age of 56 years. Her symptom at diagnosis was retinal dystrophy presenting with visual impairment. Her medical history mentioned moderate LVH with transient palpitations. She completed lower secondary education (low educational level), is married and has three healthy children. She has a completely normal cognitive functioning (Table 2). Momentarily she is on sick-leave since she is unable to work and lost her driver’s license following visual impairment. She has no dysmorphic features (Fig. 1f).

##### Patient 3

This female was diagnosed at the age 62 years. She was already known with asymptomatic HCM after a routine electrocardiography checkup. After diagnosis of MPS III she was diagnosed with asymptomatic retinal dystrophy. She has mild hepatomegaly on ultrasound. She has a Bachelor of Science degree in teaching (high educational level) and works as a primary school teacher. She refused neurocognitive testing since she experiences no issues with her cognition. She is married and has five healthy children. She has her driver’s license. She has no dysmorphic features.

##### Patient 4

This patient was diagnosed at the age of 51 years. At diagnosis he expressed visual impairment after which retinal dystrophy was diagnosed, in addition to mild asymptomatic LVH and mild hepatomegaly. He completed lower secondary education (low educational level) and runs his own company. He refused formal cognitive testing as he was too busy. He has his driver’s license. He has no dysmorphic features.

##### Patient 5

This patient was diagnosed at the age of 63 years. Her medical history mentioned moderate HCM and she was diagnosed with asymptomatic mild retinal dystrophy following family screening. She completed upper secondary school (intermediate educational level) and works as a secretary. She is married and has three healthy children. She has a completely normal cognitive functioning (Table 2). She has her driver’s license. She has no dysmorphic features.

#### Family 2

In this family 2 siblings are affected who were both diagnosed as part of family screening. The index patient was a nephew with a classic progressive phenotype who was therefore not included in this cohort.

##### Patient 6

This male was diagnosed at the age of 5 years. His mother insisted on metabolic testing for MPS III as she felt that his school performance did not meet the expected level based on the educational level of his parents, and MPS IIIA was diagnosed in his nephew (slowly progressing phenotype, (p.(Arg245His) / p.(Ser298Pro)). Despite the fact that the consulted experienced metabolic pediatrician did not observe any signs or symptoms leading to a suspicion of MPS III, urinary GAG screening was performed. His highest completed education was middle vocational education (intermediate education level). He is currently 21 years old, has a supervised job and lives with his parents. He studied for his driver’s license but failed for the theoretical exam. He has an impaired processing speed on neurocognitive testing (Table 2). He wears glasses for a refractive error leading to good vision. A cardiac ultrasound showed no abnormalities. He has no dysmorphic features (Fig. 1a).

##### Patient 7

This male was tested at the age of 3 years in the absence of any signs or symptoms of MPS III as part of family screening. He is now 19 years old and completing his last year of middle vocational education which corresponds with an intermediate educational level. He has a normal cognitive functioning (Table 2). He has no vision problems and a cardiac ultrasound showed no abnormalities. This patient has no dysmorphic features (Fig. 1b).

#### Family 3

In this family 2 siblings are affected. Patient 8 is the index patient. Patient 9 was diagnosed as part of family screening.

##### Patient 8

This female patient was diagnosed with MPS IIIA by WES, at the age of 49 years, after evaluation of retinal dystrophy by the ophthalmologist (L.I.B.) involved in the diagnosis of family 1. Further studies showed asymptomatic but severe HCM and mild hepatomegaly on ultrasound. She completed upper secondary school (intermediate educational level). She is currently on sick leave with visual impairment and she can no longer drive. She has no cognitive complaints and has a normal cognitive functioning (Table 2). She is married and has a healthy son. She has no dysmorphic features.

##### Patient 9

This patient was diagnosed at the age of 41 years following family screening. She had no medical history but reported some visual impairment and was diagnosed with retinal dystrophy after the diagnosis of MPS III, in addition to mild LVH. She has a Bachelor of Science degree (high educational level) and runs her own company. She refused neurocognitive testing as she had no cognitive complaints. She has two healthy children. She has her driver’s license. She has no dysmorphic features.

#### Family 4

In this family 2 siblings are affected. Patient 10 was diagnosed as part of family screening. The index patient, her 5 year older sister, had followed special education from the age of 8 years and had progressive cognitive decline leading to diagnostic studies by WES. Therefore, the index patient has not been included in this cohort.

##### Patient 10

This female patient was diagnosed at age 27 with MPS IIIB following family screening. At the time of diagnosis patient 10 functioned with a stable, mild neurocognitive delay (Table 2). Her highest completed level of education is middle vocational education (intermediate education level). She is currently 34 years old and has a supervised job. She lives in an assisted living facility. She has no abnormalities on cardiac ultrasound and no vision complaints. She has no dysmorphic features (Fig. 1c).

#### Family 5

In this family 2 siblings are affected. His older brother has a more severe phenotype and was therefore not included in this cohort.

##### Patient 11

This male patient was diagnosed with MPS IIIA by WES, following a decline in neurocognitive impairment at the age of 41 years which became apparent after a deterioration in daily activities. He was diagnosed with retinitis pigmentosa following impairment of night vision in his late teens. He had successfully completed comprehensive secondary school (year 11–16) but did not sit final exams. He lived with his parents until the age of 37. After this, he lived in an assisted living accommodation. He was able to travel independently until the age of 37. He has some mild dysmorphic characteristics which may fit the diagnosis of MPS III (Fig. 1d).

#### Family 6

In this family there is 1 patient included, the index patient.

##### Patient 12

This patient was diagnosed at the age of 68 with symptomatic moderate HCM. Metabolic testing was performed after an unremarkable gene panel for HCM, and led to the diagnosis of MPS IIIA. Her medical history mentioned unexplained visual impairment at age 59, but was later diagnosed as retinal dystrophy. She has completed lower vocational education (low educational level) and has worked until her retirement. She is married and has two healthy children. Neurocognitive testing was not performed since she had no cognitive complaints. She has no dysmorphic features.

#### Cases from the literature

Previously, as far as we know, two case reports have been published reporting on MPS III patients with comparably mild phenotypes (Table 1). The first case report describes a MPS IIIA patient who presented at the age of 53 years with a hypertrophic cardiomyopathy without any neurocognitive problems [16]. The diagnosis was established after an endomyocardial biopsy which revealed storage vacuoles with acid mucopolysaccharides. This patient worked as a schoolteacher. Another report describes two sisters with MPS IIIC, who were asymptomatic until their third decade of life [17]. They were diagnosed at ages 42 and 46 years because of adult onset dementia and retinitis pigmentosa. Both had followed normal secondary education.

## Discussion

We report 12 adult patients from 6 families with an unusual presentation of MPS III. In contrast to patients with the classical phenotype of MPS III, these patients showed a remarkable late-onset and mild cognitive impairment, and some patients with a non-neuronopathic phenotype consisting of retinal dystrophy and/or HCM. This indicates that these and the three previously reported cases with such an attenuated phenotype are all part of a mild- or non-neuropathic phenotype of MPS III [16, 17]. All twelve patients completed a normal secondary education. At last follow-up, at a median age of 47 years, only three patients (6.2, 10.4, 11.5) had a mild neurocognitive impairment including one with a slow decline after the age of 41 years. All other patients have a normal cognitive functioning**.** Only one patient has mild facial coarsening which is characteristic for MPS III; all others patients have normal features.

Before diagnosis, visual impairment due to retinal dystrophy was present in four patients and symptoms due to cardiomyopathy also in four patients. One of these patients had a combination of clinical symptoms due to both retinal dystrophy and cardiomyopathy. After diagnosis of MPS III, retinal dystrophy and hypertrophic cardiomyopathy were detected in nine and eight patients respectively. While none of these symptoms has been reported as a presenting symptom in patients with the more common rapid progressing phenotype, both retinal dystrophy [25, 26] and LVH and HCM [27, 28] have been reported to occur during the course of the disease in patients with both phenotypes. Pericentral retinitis pigmentosa, a specific subtype of retinal dystrophy, has been observed as sole symptom in association with mutations in the *HGSNAT* gene, encoding the lysosomal enzyme heparin-alpha-glucosaminide N-acetyltransferase a deficiency of which causes MPS IIIC (OMIM #252930) [29, 30]. Unfortunately, neither urinary GAG levels nor the activity of the involved enzyme (were investigated or reported in these studies. We now show that both retinal dystrophy with severe visual impairment and clinically relevant hypertrophic cardiomyopathy can be the only presenting symptoms in MPS IIIA. We hypothesize that prolonged exposure to slowly accumulating HS may lead to retinal degeneration and cardiomyopathy. Indeed, a recent study showed subclinical left ventricular dysfunction, assessed by speckle-tracking echocardiography, in patients with the rapidly and slowly progressing phenotypes [31].

In eight patients in this study cognitive testing had recently been performed. In four of them there were no signs of cognitive impairment and cognitive assessment was done only because of the diagnosis of MPS III. Three patients showed remarkable disharmonic profiles generally in favor of verbal comprehension with very low processing speed. Disharmonic profiles have previously been described in more severely affected MPS III patients [32].

While significant sibling disparity has not been reported in MPS III, we observed striking differences between patient 10.4, with mild cognitive problems in adulthood, and her older sibling who had progressive loss of cognitive function from the age 8 years. Patient 11.5 also has a sibling with a more classical course of the disease who is now non-ambulant and fully care dependent. Probably, other (epi) genetic and/or environmental factors influence the course of the disease more profoundly in patients with genotypes that may convey a very mild phenotype than in those that convey a more severe phenotype.

Our study has limitations, as only patients attending one of the participating centers were included. We thus cannot estimate the prevalence of the mild- and/or non-neuropathic MPS III phenotypes in the population. Although we cannot exclude founder effects causing the occurrence of these remarkably mild phenotypes in the Netherlands and the UK, we feel that the wide availability of WES for clinical diagnostic purposes in these countries is the most important factor for diagnosis. We therefore expect that these patients are not unique and confined to our countries, and that MPS III patients with a similar mild- or non-neuropathic phenotype will often miss proper diagnosis. Even when WES is used as diagnostic strategy, MPS III might well be missed, as WES panels generally only include genes known to be involved in specific conditions such as cardiomyopathy or retinal degeneration, and these panels will most often not include MPS III genes. Based on our study, we feel that WES data extraction for these indications should be expanded with MPS III genes when the targeted gene panel if not conclusive.

It is important to diagnose mild - and non-neuronopathic MPS III patients for several reasons. First, such a diagnosis will allow monitoring for potential additional complications, such as for HCM in patients with retinal dystrophy as a first symptom and vice versa. Second, a diagnosis of MPS III allows for genetic counseling of relatives, thus enhancing reproductive autonomy [33]. Third, a phase IIB study in MPS IIIA patients concluded that intrathecal enzyme replacement therapy (ERT) did not sufficiently alter CNS disease, but that ERT may have somatic efficacy [34]. Intravenous ERT might be a successful treatment in MPS III patients with cardiomyopathy, as ERT was demonstrated as effective by reducing left ventricular mass index in children with Pompe disease [35]. Furthermore, a number of disease modifying therapies are currently under investigation for the CNS disease of MPS III, and some are already in clinical trial including both gene therapy and ERT [36–40]. Patients with a very slow evolution of disease may well be even more responsive to treatment than the patients with the classical rapidly progressing phenotype, as the therapeutic window in this latter group is small [1]. However, it will be very difficult, if not impossible, to assess treatment efficacy in patients with such slowly progressing phenotypes by clinical evaluation and this can probably only be done by following a biomarkers response. Unfortunately, to date no biomarker for MPS III, correlating with disease progression, has been identified.

## Conclusions

In conclusion, MPS III can present at adult age with a remarkably mild and late onset neurocognitive impairment or even non-neuronopathic somatic phenotype with either retinal dystrophy or hypertrophic cardiomyopathy. Awareness of this phenotype is essential as patients and families can benefit from diagnosis as this leads to appropriate diagnostic strategies, monitoring, family counseling and, hopefully within the next decades, treatment. We strongly advise to add MPS III genes as a second diagnostic panel in targeted gene panels for retinal dysfunction and cardiomyopathy.

## Data Availability

All data analyzed are included in this article.

## Abbreviations

GAGs: Glycosaminoglycans
HCM: Hypertrophic cardiomyopathy
HS: Heparan sulfate
LVH: Left ventricle hypertrophy
MPS: Type III: mucopolysaccharidosis type III
RD: Retinal dystrophy
